# Increased mitochondrial respiration of adipocytes from metabolically unhealthy obese compared to healthy obese individuals

**DOI:** 10.1038/s41598-020-69016-9

**Published:** 2020-07-24

**Authors:** Anja Böhm, Michaela Keuper, Tobias Meile, Marty Zdichavsky, Andreas Fritsche, Hans-Ulrich Häring, Martin Hrabě de Angelis, Harald Staiger, Andras Franko

**Affiliations:** 1 Department of Internal Medicine IV, Division of Endocrinology, Diabetology, Nephrology, University Hospital Tübingen, Eberhard Karls University Tübingen, Otfried-Müller-Str. 10, 72076 Tübingen, Germany; 2 German Center for Diabetes Research (Deutsches Zentrum für Diabetesforschung, DZD), Neuherberg, Germany; 3 Institute for Diabetes Research and Metabolic Diseases of the Helmholtz Center Munich At the Eberhard Karls University Tübingen, Tübingen, Germany; 4 Department of Molecular Bioscience, The Wenner-Gren Institute, Stockholm University, Stockholm, Sweden; 5 Clinic for General, Visceral, Thoracic and Transplant Surgery, Klinikum Stuttgart, Bad Cannstatt, Germany; 6 Department of General, Visceral and Transplant Surgery, University Hospital, Eberhard Karls University Tübingen, Tübingen, Germany; 7 Interfaculty Centre for Pharmacogenomics and Pharma Research at the Eberhard Karls University Tübingen, Tübingen, Germany; 8 Institute of Experimental Genetics, Helmholtz Zentrum München, German Research Center for Environmental Health, Neuherberg, Germany; 9 Chair of Experimental Genetics, Technical University München, Freising-Weihenstephan, Germany; 10 Institute of Pharmaceutical Sciences, Department of Pharmacy and Biochemistry, Eberhard Karls University Tübingen, Tübingen, Germany

**Keywords:** Mechanisms of disease, Obesity, Metabolic syndrome, Obesity, Molecular medicine

## Abstract

Among obese subjects, metabolically healthy (MHO) and unhealthy obese (MUHO) subjects exist, the latter being characterized by whole-body insulin resistance, hepatic steatosis, and subclinical inflammation. Insulin resistance and obesity are known to associate with alterations in mitochondrial density, morphology, and function. Therefore, we assessed mitochondrial function in human subcutaneous preadipocytes as well as in differentiated adipocytes derived from well-matched donors. Primary subcutaneous preadipocytes from 4 insulin-resistant (MUHO) versus 4 insulin-sensitive (MHO), non-diabetic, morbidly obese Caucasians (BMI > 40 kg/m^2^), matched for sex, age, BMI, and percentage of body fat, were differentiated in vitro to adipocytes. Real-time cellular respiration was measured using an XF24 Extracellular Flux Analyzer (Seahorse). Lipolysis was stimulated by forskolin (FSK) treatment. Mitochondrial respiration was fourfold higher in adipocytes versus preadipocytes (*p* = 1.6*10^–9^). In adipocytes, a negative correlation of mitochondrial respiration with donors’ insulin sensitivity was shown (*p* = 0.0008). Correspondingly, in adipocytes of MUHO subjects, an increased basal respiration (*p* = 0.002), higher proton leak (*p* = 0.04), elevated ATP production (*p* = 0.01), increased maximal respiration (*p* = 0.02), and higher spare respiratory capacity (*p* = 0.03) were found, compared to MHO. After stimulation with FSK, the differences in ATP production, maximal respiration and spare respiratory capacity were blunted. The differences in mitochondrial respiration between MUHO/MHO were not due to altered mitochondrial content, fuel switch, or lipid metabolism. Thus, despite the insulin resistance of MUHO, we could clearly show an elevated mitochondrial respiration of MUHO adipocytes. We suggest that the higher mitochondrial respiration reflects a compensatory mechanism to cope with insulin resistance and its consequences. Preserving this state of compensation might be an attractive goal for preventing or delaying the transition from insulin resistance to overt diabetes.

## Introduction

Obesity and type 2 diabetes have become important health burdens of the twenty-first century in western, and most recently also in developing countries. Until now, several pathomechanisms were discovered underlying the development of insulin resistance, but our knowledge is still incomplete.

Reduced muscle oxidative capacity is known to correlate with insulin resistance^1^. In adipose tissue, mitochondrial content might be reduced in obesity^2–5^ and obese diabetic subjects^6^. Additionally, reduced mitochondrial activity was shown in obesity^5,7,8^ and type 2 diabetes^7,9^. Independent of obesity, electron transport chain genes were reduced in visceral adipose tissue from women with type 2 diabetes compared to healthy controls^10^.

Contrarily, obese individuals with diabetes demonstrated unchanged oxygen flux per mitochondrial content in adipose tissue biopsies, compared to non-diabetic, obese controls^9^, and mitochondrial content was not altered between obese and obese, diabetic individuals^7,9^. Although a correlation of adipose tissue mitochondrial function and diabetes and/or obesity is convincing, the discrimination to either diabetes or obesity is even more complex.

Additionally, ´mitochondrial (dys)function` often refers to various processes^11,12^, and it is crucial to discriminate precisely between mitochondrial content, morphology, respiration and other items, e.g., dynamics, turnover, and plasticity^12^.

To develop individual, effective prevention strategies against obesity-related insulin resistance, the molecular basis of metabolic subphenotypes must be decoded. In the obese state, metabolically healthy (MHO) and metabolically unhealthy obesity (MUHO) are known^13^; the latter, affecting ~ 70% of obese subjects, is characterized by whole-body insulin resistance, ectopic lipid deposition, and subclinical inflammation^14,15^.

Aim of this study was, to investigate the preadipocyte- and adipocyte-specific mitochondrial function in MUHO subjects compared to MHO, providing further explanations for the development of insulin resistance and/or metabolic dysfunction. Therefore, the mitochondrial respiration of isolated human subcutaneous preadipocytes and in vitro differentiated adipocytes derived from insulin-resistant (MUHO) versus (vs.) insulin-sensitive morbidly obese (MHO) subjects, matched for sex, age, BMI, and percentage of body fat, was determined under basal and forskolin (FSK)-stimulated conditions.

## Materials and methods

### Human preadipocyte donors and phenotyping

4 insulin-resistant versus 4 insulin-sensitive (based on insulin sensitivity index), non-diabetic, morbidly obese Caucasians (BMI > 40 kg/m^2^) were matched for gender, age, BMI, and percentage of body fat (Table 1). All participants underwent physical examination and routine laboratory tests. Informed written consent was given by all individuals. The study protocol has been approved by the ethics committee of the Tübingen University and was in accordance with the declaration of Helsinki. Metabolic phenotyping was done by a 2-h (5-point) 75 g oral glucose tolerance test (OGTT). Routine laboratory tests were performed on ADVIA 1,800 clinical chemistry system (Siemens healthcare systems, Erlangen, Germany). Leptin and adiponectin were measured by ELISA (R&D systems, Wiesbaden, Germany). Percentage of body fat was measured by bioelectrical impedance (BIA-101, RJL Systems, Detroit, USA).

**Table 1:** Data represent number (N) or means ± standard deviation.

	MHO	MUHO	p1	p2
N (women/men)	4 (2/2)	4 (2/2)	–	–
Age (y)	46 ± 6	44 ± 13	–	–
BMI (kg/m^2^)	48.5 ± 4.4	50.0 ± 2.5	0.8	–
Body fat (%)	39.4 ± 12.5	42.7 ± 10.7	0.6	–
AUC_Glucose 0–120_ (mmol/L)	17.2 ± 1.6	21.0 ± 2.2	0.0455	0.08
AUC_Insulin 0–120_ (pmol/L)	956 ± 382	3,091 ± 382	0.0025	0.0065
ISI OGTT (10^19^ L^2^/mol^2^)	8.46 ± 2.93	2.61 ± 0.60	0.0016	0.0049
Free fatty acids (µmol/L)	667 ± 332*	787 ± 118	0.4	0.5
Leptin (ng/mL)	54.5 ± 28.4*	57.9 ± 13.8	0.8	0.9
Adiponectin (µg/mL)	9.13 ± 4.30*	6.01 ± 3.24	0.09	0.2

Prior to statistical analysis, data were log_e_-transformed in order to approximate normal distribution and adjusted; p1—p-value after adjustment for gender and age; p2—*p*-value after adjustment for sex, age, and BMI. AUC—area under the curve; BMI—body mass index; ISI—insulin sensitivity index; OGTT—oral glucose tolerance test; MHO—metabolically healthy obese subjects; MUHO—metabolically unhealthy obese subjects. *Due to limited sample amount, measurements of three probands only.

### Calculations

The insulin sensitivity index (ISI) was calculated using the method of Matsuda and DeFronzo, (10,000/square root of [fasting glucose × fasting insulin × mean glucose × mean insulin])^16^. The areas under the curves (AUCs) of glucose and insulin levels during the OGTT were calculated according to the trapezoid method.

### Human subcutaneous preadipocytes

Human subcutaneous fat biopsies were obtained during gastric sleeve surgery after an overnight fast. Preadipocytes were isolated as previously described^17^ and differentiated in vitro to adipocytes according to our in-house protocol^18^. Other than stated there, cell culture for mitochondrial respiration measurement was performed on XF24 V7 Cell Culture Microplates (Agilent Technologies, USA). The differentiation process was verified using light microscopy.

### Determination of cellular mitochondrial function

**Figure 1:**
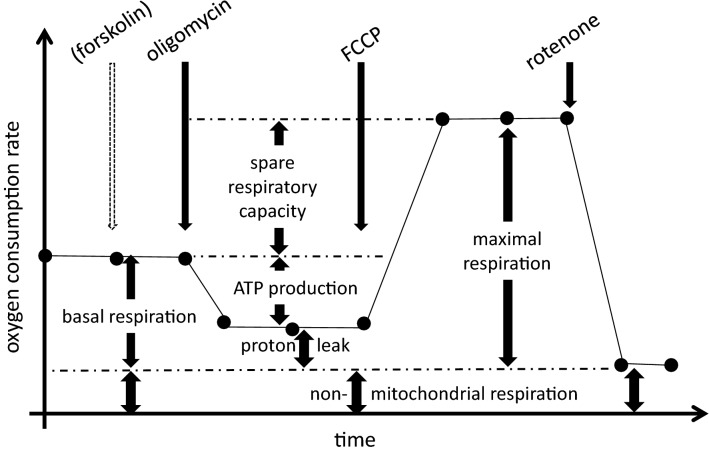
Schematic representation of the mitochondrial stress test as adopted from Seahorse (Agilent Technologies) as well as^19,53^. Dotted arrow: optional treatment with 5 µM forskolin (FSK). FCCP: Carbonyl cyanide 4-(trifluoromethoxy)phenylhydrazone; MHO: metabolically healthy obese subjects; MUHO: metabolically unhealthy obese subjects. For analysis, non-mitochondrial respiration was subtracted from all results.

Metabolic flux analyses were performed in preadipocytes (day 0) and differentiated (day 20) adipocytes using an XF24 Extracellular Flux Analyzer (Agilent Technologies). Basal, uncoupled, and non-mitochondrial cellular O_2_ consumption rates (OCR), proton leak, and estimates of anaerobic glycolysis (extracellular acidification rates [ECAR]) were measured according to manufacturer’s protocol and Yehuda-Shnaidman et al.^19^, using DMEM assay medium containing 17.5 mM glucose, 1 mM pyruvate, pH7.4. After equilibration, a mitochondrial stress test was performed with oligomycin (1 µM; inhibition of ATP-synthase; provides an estimate for ATP production), FCCP (Carbonyl cyanide 4-(trifluoromethoxy)phenylhydrazone; 0.6 µM; uncoupling/maximal respiration) and rotenone (3 µM; inhibition of complex I; approximately non-mitochondrial respiration). Non-mitochondrial respiration was subtracted from all results. For experimental setting of the stress test, see Fig. 1. For detailed FCCP titration experiments, see supplemental Fig. S6. Results were normalized for cellular protein content (measured by RC-DC-Assay from BioRad, California, USA) after measurement of cellular respiration and are therefore given in arbitrary units (AU), corresponding to pmol O2 per minute per µg protein. To test mitochondrial responsiveness, lipolysis was stimulated by forskolin (FSK; 5 µM) treatment for 45 min after basal measurement as a separate OCR measurement. Respiratory coupling ratio was assessed by FCCP/oligomycin ratio.

### Real time RT-PCR

Cells were grown in 6-wells as described before^18^. At day 20 of differentiation, cells were washed once with PBS, and lysed with 700 µl QIAzol. RNA isolation (miRNeasy Kit, Qiagen, Hilden, Germany) and cDNA synthesis (Transcriptor First Strand cDNA Synthesis Kit, Roche Diagnostics, Mannheim, Germany) were performed according to manufacturers’ protocols using random hexamer primers. Real time RT-PCR was done on LightCycler 480 (Roche Diagnostics). Primers were designed using the Probe Design Library (Roche Diagnostics) and purchased from TIB MOLBIOL (Berlin, Germany), probes were purchased from Roche Diagnostics. For primer sequences, see table S1 in supplements. All RNA data are presented relative to the expression of the housekeeping gene RPS13 using the ∆∆Ct method.

### Lactate measurement

Lactate levels were determined in conditioned media from adipocytes seeded in 6-well plates as published before^18^. Samples were diluted 1:4 in 0.9% sodium chloride solution and measured on the ADVIA 1,800 (Siemens healthcare systems).

### Citrate synthase (CS) activity

CS activity was measured by a kit (“Citrate Synthase Activity Assay Kit”, ab119692; Abcam, Cambridge, UK) according to manufacturer’s protocol and normalized for protein. The cells were seeded and differentiated until day 16 in 10 cm-dishes.

### Western blots

Protein concentration of cell lysates was determined by Nanodrop (ThermoFisher Scientific, Waltham, USA); 35 µg protein/lane were separated by sodium dodecyl sulfate polyacrylamide gels in various concentrations as follows: PGC1α and SDHA by 7.5%, UQCRC2 10%, and TFAM by 12.5%. Proteins were transferred to a PVDF membrane. Ponceau Red and tubulin served as loading controls. Antibodies were purchased from Abcam, Cambridge, UK (PGC1α and UQCRC2) and Cell Signaling, Danvers, USA (SDHA TFAM, and tubulin). Imaging was performed with ChemiDoc Touch (BioRad, Hercules, USA).

### Reactive oxygen species (ROS)

H_2_O_2_ concentration was measured in cell lysates using assay STA-844 (“OxiSelect Hydrogen Peroxide/Peroxidase Assay Kit”, Cell Biolabs San Diego, USA) according to manufacturer’s protocol and protein-normalized.

### cAMP concentration

cAMP concentration was measured in cell lysates by an ELISA (ab133038 acetylated version, “cyclic AMP direct ELISA kit”, Abcam, Cambridge, UK). No protein-normalization was applied.

### Lipolysis

Cells were seeded in triplicates on 6-well plates and differentiated until day 16. After starving for 24 h, cells were incubated for 2 h with Krebs Ringer bicarbonate + 1% bovine serum albumin + 5 µM FSK. Supernatant was pooled from 3 wells, afterwards glycerol concentration was determined in duplicates with Free Glycerol Assay Kit (#65,337, Abcam) according to manufacturer’s instructions.

### Statistics

Statistics was performed with a two-sided homoscedastic t-test, if not stated otherwise. If adjusted, a multiple linear regression analysis was performed. A *p*-value < 0.05 was considered as statistically significant. Software packages: JMP 11 (SAS Institute Inc., USA) and Excel 2010 (Microsoft, Unterschleißheim, Germany). FCCP data from one participant were extremely high. After classification as an outlier with Grubbs’ test (using: https://graphpad.com/quickcalcs/grubbs1/), these FCCP data were excluded. Raw data in the text are presented as mean ± standard deviation.

## Results

### Anthropometric and metabolic characterization of the cell donors

All subjects were middle-aged and morbidly obese (Table 1). Fifty percent of the cell donors, both in the MHO and MUHO group, were women (Table 1). After adjustment for gender and age, insulin sensitivity (insulin sensitivity index, ISI), glucose tolerance (AUC_glucose_), and the insulin response to oral glucose intake (AUC_insulin_) were significantly different between MHO and MUHO (Table 1). After additional adjustment for BMI, the differences in ISI and AUC_insulin_ remained significant (Table 1). As intended by matching, the groups did not significantly differ in age (p_unadjusted_ = 0.7), body fat, or BMI (Table 1). Notably, no significant differences were detected regarding free fatty acids (FFAs; Table 1). Habitual physical activity score^20^ was not different between the groups (MHO vs. MUHO; 7.6 vs. 6.8; p_Wilcoxon_ = 0.4).

### Mitochondrial respiration of preadipocytes

There was no significant difference in oxygen consumption between MHO compared to MUHO preadipocytes (basal respiration, MHO vs. MUHO, 1.02 ± 0.69 vs. 1.01 ± 0.47, *p* = 1.0). Regarding ECAR as a rough estimate of glycolysis, there was no difference between both preadipocyte groups either (basal state, MHO vs. MUHO, 0.33 ± 0.04 vs. 0.42 ± 0.1, *p* = 0.18).

### Mitochondrial respiration of adipocytes

In differentiated adipocytes, there was a fourfold increased mitochondrial respiration in comparison to preadipocytes (whole group basal respiration in preadipocytes: 1.1 ± 0.4, in adipocytes: 4.2 ± 1.3, *p* = 1.6*10^–9^). No significant differences in ECAR were found between preadipocytes and adipocytes (0.4 ± 0.1 vs. 0.5 ± 0.3, *p* = 0.2).

**Figure 2:**
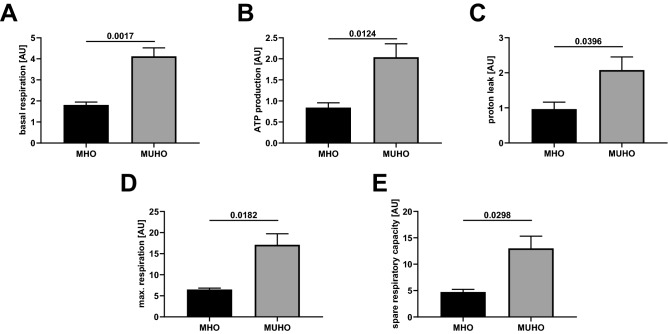
Mitochondrial respiration in differentiated adipocytes, n = 4/group; mean ± SEM; panel A—basal respiration; B—ATP production (oligomycin subtracted from basal, see also Fig. 1); C—proton leak (oligomycin); D—maximal respiration (FCCP), n = 3 versus 4; E—spare respiratory capacity (basal subtracted from FCCP).

Marked group-specific differences in mitochondrial oxygen consumption were found between MUHO versus MHO adipocytes (Fig. 2): basal respiration (Fig. 2A) and ATP production (2B) were significantly elevated in MUHO. Also, proton leak (2C), evidenced by oligomycin application, as well as maximal respiration (2D) were higher in MUHO. Accordingly, spare respiratory capacity (SRC, 2E), roughly displaying adaptation to metabolic changes, was elevated in MUHO. On the other hand, no differences were detected in the respiratory coupling ratio, which mirrors membrane coupling (MUHO vs. MHO, 8.4 ± 1.1 vs. 7.6 ± 3.6, *p* = 0.7).

### Glycolytic flux of adipocytes

ECAR, an estimate for anaerobic glycolysis, did not show any difference between adipocytes groups under all conditions (e.g., basal ECAR, MUHO vs. MHO, 0.5 ± 0.2 vs. 0.6 ± 0.4, *p* = 0.8). In addition to ECAR values, lactate levels as approximation of anaerobic glycolysis were determined: no differences in cell culture supernatants between MUHO and MHO (MUHO vs. MHO, 4.1 ± 0.8 mmol/l vs. 4.8 ± 0.8 mmol/l, *p* = 0.3) were detected.

### FSK-induced alterations in mitochondrial respiration

Lipolysis was stimulated with FSK resulting in fuel supply for the cell. As expected, FSK did not change mitochondrial respiration in preadipocytes (data not shown). In adipocytes, FSK augmented basal respiration (FSK vs. control, 6.9 ± 2.2 vs. 4.4 ± 1.2, *p* = 0.01), and this was observed in both MHO and MUHO (FSK vs. control, MHO: 5.0 ± 1.4 vs. 3.2 ± 0.8, *p* = 0.02; MUHO: 8.7 ± 1.7 vs. 5.1 ± 0.8, *p* = 0.0004). Additionally, proton leak increased after FSK stimulation from 1.5 ± 0.8 to 6.1 ± 2.4 (*p* = 0.0002), also in both groups: in MHO, from 1.0 ± 0.4 to 4.3 ± 2.0 (*p* = 0.02); in MUHO, from 2.1 ± 0.8 to 7.8 ± 1.4 (*p* = 0.0003). Respiratory coupling ratio was accordantly reduced (control vs. FSK, 8.1 ± 2.2 vs. 2.8 ± 1.1, *p* < 0.0001), significantly in MUHO adipocytes (control vs. FSK, 8.4 ± 1.1 vs. 2.2 ± 0.5 vs., *p* < 0.0001), and by trend in MHO (control vs. FSK, 7.6 ± 3.6 vs. 3.5 ± 1.3, *p* = 0.08).

**Figure 3:**
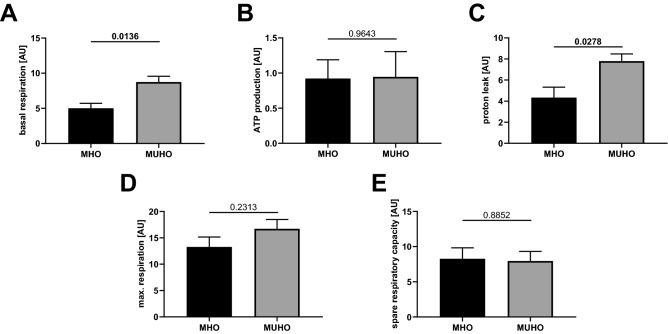
Mitochondrial respiration in differentiated adipocytes after FSK injection, n = 4/group, if not stated otherwise; mean ± SEM; panel A—basal respiration; B—ATP production (oligomycin subtracted from basal, see also Fig. 1); C—proton leak (oligomycin); D—maximal respiration (FCCP); E—spare respiratory capacity (basal subtracted from FCCP), n = 3 versus 4.

Thus, stimulated by FSK, mitochondrial respiration in MHO and MUHO adipocytes still differed with respect to basal respiration (Fig. 3A) and proton leak (3C), whereas the elevated ATP production (3B), maximal respiration (3D), and spare respiratory capacity (3E) in MUHO adipocytes now was blunted by FSK. Thus, FSK was able in MHO adipocytes only to increase maximal respiration even more (control vs. FSK, MHO: 6.5 ± 0.5 vs. 13.3 ± 3.7, *p* = 0.03; MUHO: 17.1 ± 5.2 vs. 16.7 ± 3.5).

### Gene expression analysis

To study whether altered expression of metabolic genes could account for the elevated mitochondrial respiration in MUHO adipocytes, mRNA expression of lipogenic (Supplemental Fig. S1A), lipolytic (S1B), β-oxidative (S1C), or oxidative phosphorylation (OXPHOS, S1D) genes were analyzed by RT-qPCR. However, no differences were found between MUHO and MHO groups. Regarding master regulators of mitochondrial biogenesis (S1E), we found an elevated expression of TFAM in MUHO adipocytes, while other markers for biogenesis (NRF1, NRF2, ERRα, PGC1α) did not differ between the groups. Additionally, no differences in protein levels of selected genes were found (Fig. S2), neither differences in mitochondrially encoded genes (Fig. S3).

### Additional investigation of underlying pathomechanisms

Citrate synthase activity, as an accepted estimate of mitochondrial number, revealed no group differences, neither in pre- nor in adipocytes (Fig. S1F).

Also, no differences in lipolysis (measured by glycerol release in supernatant of adipocytes), cAMP levels (determined in cell lysates of adipocytes), or reactive oxygen species (ROS) production (measured by H_2_O_2_ concentration in cell lysates) between MHO and MUHO were found under the applied culture conditions (Fig. S4).

**Figure 4:**
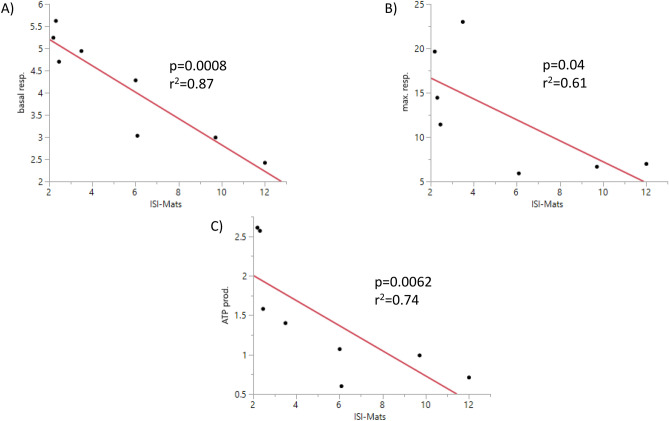
Correlation of ISI_Matsuda_ and A) basal respiration, B) maximal respiration (n = 7), and C) ATP production in arbitrary units. n = 8, if not stated otherwise. Red line: regression line; analysis by ANOVA.

However, a negative linear correlation of in vitro mitochondrial respiration to cell donors’ insulin sensitivity was successfully shown for basal respiration, maximal respiration, and ATP production (Fig. 4).

## Discussion

As anticipated due to immaturity of preadipocytes, our data showed that preadipocytes had limited mitochondrial respiration, which was already shown in mouse and human adipocytes^21–23^. Thus, the marked increase in mature adipocytes’ respiration was now confirmed in human adipocytes of MUHO and MHO subjects.

To our knowledge, this is the first study that analyzed mitochondrial respiration in human adipocytes of morbidly obese, non-diabetic individuals with divergent insulin-sensitivity. We observed an unexpected elevated mitochondrial respiration in MUHO adipocytes (basal respiration, ATP production, proton leak, uncoupled respiration and spare respiratory capacity). The higher mitochondrial respiration was not due to different mitochondrial content estimated by citrate synthase activity. Thus, our data point to an increased respiration per mitochondrion, although mRNA levels for oxidative phosphorylation were not altered among groups.

As postulated by Randle^24^, fatty acid metabolism and glucose metabolism are mutually controlled and an altered ratio of β-oxidation/glycolysis-derived ATP-production might be a hypothesis for differences in MHO versus MUHO. Indeed, under hypoglycemic conditions adipocytes switch from glycolytic to β-oxidative ATP production^22^. However, in our study, where glucose and pyruvate both were abundant, we did not find hints for an altered glycolysis as measured by ECAR and lactate levels. In obese and/or diabetic individuals a ‘metabolic inflexibility’ was described, meaning an impaired fuel switch from fatty acid to glucose oxidation at least in skeletal muscle^25^. Based on that potentially impaired switch but equal glycolysis, a relatively increased β-oxidation in MUHO might be responsible for enhanced mitochondrial respiration. Additionally, in MUHO the insulin-mediated suppression of lipolysis should be limited due to insulin-resistance, likewise promoting enhanced lipolysis and fuel surplus. However, there was neither a difference in circulating FFAs in the cell donors, nor did we detect any differences regarding glycerol secretion at a cellular level under the applied culture conditions. Also, FSK leads to lipolysis as well as to cAMP accumulation^26^, and cAMP in turn was shown to increase uncoupling^19^. Though, altered cellular cAMP-levels, (which were, however, not protein-normalized) underlying the elevated respiration in MUHO were excluded; this additionally supports no lipolytic differences among the groups.

Mitochondrial respiration seems to be highly regulated by ATP demand^27^. Thus, an increased ATP demand of MUHO adipocytes might play a role, although this presumption will need further studies. Without an increase in ATP demand in MUHO, an accumulation of ROS and increased ER-stress might occur in MUHO. This in turn could promote insulin resistance^28,29^, resulting in one possible pathomechanism for the progression to diabetes. A contrary point of view is that augmented ROS production at least in mice could represent an adaptive mechanism to prevent obesity^30^. However, no difference in ROS production (measured by H_2_O_2_ level) was detected between MUHO and MHO adipocytes. In this context it must be mentioned that the level of ROS measured in the cell might be almost unchanged, while mitochondrial ROS synthesis might be increased, e.g., due to activity of antioxidant mechanisms. Furthermore, novel studies suggest, that mitochondrial ROS production could also be regulated by succinate, proline dehydrogenase and superoxide dismutase^31^, which however were not investigated in our study.

Interestingly, not only basal respiration, ATP production and proton leak were elevated in MUHO adipocytes, but—after forced uncoupling by FCCP—also maximal respiration and moreover spare respiratory capacity were higher. This might imply that MUHO adipocytes are more prone to deal with high amounts of fuels and thus might reflect a compensatory regulation. Of note, basal serum FFAs were not different between our groups. There is evidence that fatty acid spill-over from adipocytes to liver, muscle, and pancreas leads to insulin resistance^32,33^. Thus, elevated fuel consumption might protect from this spill-over.

Since MUHOs’ adipocytes seem to compensate for increased fuels compared to MHO, mitochondrial response after forced induction of lipolysis by FSK was investigated; subsequent to endogenous fuel increase, both groups were able to raise basal mitochondrial respiration significantly. In human adipocytes, increased respiration after beta-adrenergic stimulation, including the ability to uncouple, was already shown^19^. This increment is due to a reduced membrane potential^34^ and it is likely, that fatty acids serve as both, substrate and uncoupler, in adipocytes^19^. Also a reduced respiration of adipocytes from obese versus lean individuals was demonstrated, but only when stimulated by catecholamines^19^. Additionally, adiposity correlates with diminished catecholamine responsiveness of adipocytes^35^, possibly due to decreased β2-receptor density^36^. Nevertheless, in our selected equally obese but divergent insulin-resistant participants, the elevated basal mitochondrial respiration in MUHO persisted under FSK stimulation.

On the other hand, elevation of maximal respiration and therefore uncoupling by FSK was exclusively effective in MHO adipocytes, which may be due to the inability of MUHO adipocytes to increase respiration anymore (ceiling effect). Concordantly, FSK evened out the inter-group differences regarding ATP production, maximal respiration, and spare respiratory capacity, also reflecting a limit yet reached in MUHO adipocytes.

Earlier, we reported higher local levels of arachidonic acid metabolism in MUHO adipocytes^18^. In rat cardiomyocytes, arachidonic acid increased respiration under normoxic conditions, whereas during hypoxia arachidonic acid aggravated the hypoxia-related inhibition of respiration^37^. Arachidonate at submicromolar concentrations was shown to stimulate the steady-state proton translocation under conditions where electron transfer was unaffected^38^. An arachidonic acid dependent decrease of the respiratory activity in bovine heart mitochondria was due to a selective inhibition of complex I and III^39^. Thus, our data might provide another hint for arachidonic acid in the pathophysiology of insulin resistance and require further mechanistic studies.

Transient insulin resistance might be a mechanism recruited in the obese state to limit additional weight gain and to stabilize body mass^40^. In adipocytes, a protective mechanism via uncoupling was supposed^41^, but human data were lacking; our data support the hypothesis of a compensatory state in human adipocytes for the first time.

Our results are in line with others studies, since elevated whole body resting energy expenditure in non-diabetic subjects was reported to correlate positively with insulin resistance independently of fat mass^42^, although whole body energy expenditure comprises adipose tissue to a small extent^43^ and the contribution of adipose tissue mitochondrial function to whole-body energy metabolism is disputed^44^. But white adipocytes’ impact on energy metabolism might be due to prevention of lipotoxicity by fatty acid combustion^41^, and relevant in a long-term manner^45^.

Similarly, obese, insulin-resistant, non-diabetic subjects were shown to exhibit increased postprandial mitochondrial activity in the liver compared to obese diabetic patients^46^, also reflecting transient compensatory mechanisms due to insulin resistance before type 2 diabetes occurs.

As other underlying mechanisms, also involvement of PTP (permeability transition pore) or different membrane potential could be discussed^19^, e.g., as opened PTPs are associated with increased uncoupling^47^. Regarding that, additional studies are required. UCP1, as a prominent player for uncoupling in brown adipose tissue, does not play an apparent role in white adipose tissue. To what extent other uncoupling proteins as ANT3, PiC, UCP2, VDAC1, BAX, and ANT2/1 play a role in diverse functioning of MUHO’s versus MHO’s mitochondria, was not investigated. For example BAX was identified as a major regulator of uncoupling^47^.

Notably, the differences in mitochondrial function between MHO and MUHO were observed after several weeks of cell culture. This stability might imply a possible genetic and/or epigenetic background for these differences.

Preserving the better oxidative condition in MUHO might be an attractive goal for prevention of progress of insulin resistance. This assumes that the demonstrated elevation of mitochondrial respiration is just a transient protective mechanism and breaks down with the transition to overt diabetes. This was also suggested for liver mitochondrial adaptation in obesity with respect to progression from hepatic steatosis to steatohepatitis^48^.

The switch between MHO to MUHO is known to be regulated by hormonal alterations^13^. Several hormonal pathways including adipo- and myokines are recently described as key regulators of mitochondrial respiration in adipocytes. Estradiol was shown to activate protein kinase A, which consecutively elevated estrogen receptor alpha phosphorylation and stimulated mitochondrial function in adipocytes^49^. Additionally, irisin was demonstrated to inhibit adipogenesis and simulated the browning of white adipocytes, which is associated with increased mitochondrial biogenesis^50^. These results suggest that the observed difference in mitochondrial performance between MHO and MUHO adipocytes could be partly regulated by adipo- and myokines, which however were beyond the scope of our study.

Nevertheless, the discussion if altered mitochondrial function is cause or consequence of insulin resistance is still ongoing^11,12,51,52^ and further studies are required.

Strategies to provide higher oxidative capacity by remodeling white adipocytes into energy-dissipating brown-like adipocytes, or by recruitment of brown adipose tissue in human adults, are currently intensively investigated.

## Conclusion

This is the first study to investigate mitochondrial respiration in MHO versus MUHO adipocytes from otherwise healthy individuals, thus dissecting insulin resistance from obesity. We could clearly show an elevated adipocyte mitochondrial respiration in MUHO, supported by a negative correlation of in vivo insulin sensitivity with in vitro mitochondrial respiration. The differences were not due to altered mitochondrial content, fuel switch, or lipid metabolism. Maximal oxygen consumption levels in MUHO seemed to be reached, and thus the group-differences were blunted after FSK treatment. Our data suggest that elevated respiration of adipocytes derived from MUHO individuals might be of compensatory origin and reflect the insulin resistance of cell donors rather than accounting for it. Preserving this elevated respiration might be beneficial for decelerating the progress to diabetes.

## Supplementary information

Supplementary information
