# Supplementary material for: Increased mitochondrial respiration of adipocytes from metabolically unhealthy obese compared to healthy obese individuals
Source: Sci Rep. 2020 Jul 24;10:12407. doi: 10.1038/s41598-020-69016-9 (PMC7382448; doi:10.1038/s41598-020-69016-9)
Supplement: Supplementary file 1 — Supplementary information [file 41598_2020_69016_MOESM1_ESM.pdf]

# Increased mitochondrial respiration of adipocytes from metabolically unhealthy obese compared to healthy obese individuals

Anja Böhm, Michaela Keuper, Tobias Meile, Marty Zdichavsky, Andreas Fritsche, Hans-Ulrich Häring, Martin Hrabě de Angelis, Harald Staiger and Andras Franko

## Supplemental legends

**Figure S1:** Potential underlying pathomechanisms, n=4/group; mean ±SEM; mRNA expression normalized to RPS13 of genes pivotal for: lipogenesis (panel A), lipolysis (B), β- oxidation (C), respiratory chain enzymes of complexes I-IV (D), and mitochondrial biogenesis (E), analyzed in adipocytes. Panel F – citrate synthase activity in preadipocytes and differentiated adipocytes.

**Figure S2:** Western Blots for protein levels of selected genes, n=4/group, if not stated otherwise; relative band intensity values (mean ±SD) are normalized to tubulin; proteins include important proteins for A) Complex II, B) Complex III (n=3 vs. 4), and C+D) mitochondrial biogenesis. On the right side, representative original western blots are shown.

**Figure S3:** mitochondrial DNA encoded genes; mean ±SD; mRNA expression normalized to RPS13; n=2 vs.3; ND1 representing complex I, COI = complex IV, ATP6 = complex V.

**Figure S4:** additional pathomechanistic hypotheses; panel A) lipolysis, B) cAMP, and C) ROS; mean ±SD; each n=2 vs. 3; A) and c) normalized to protein.

**Figure S5:** Original western blot; blots in figure S2 were taken from this blot. Black framed parts correspond to figure S2.

**Figure S6:** FCCP titration experiments showing the mitochondrial stress test under various FCCP concentrations (in µM), showing that optimal conditions were used in this study; n=5.

**Table S1:** Primer sequences used for quantitative real-time PCR.

fig. S1

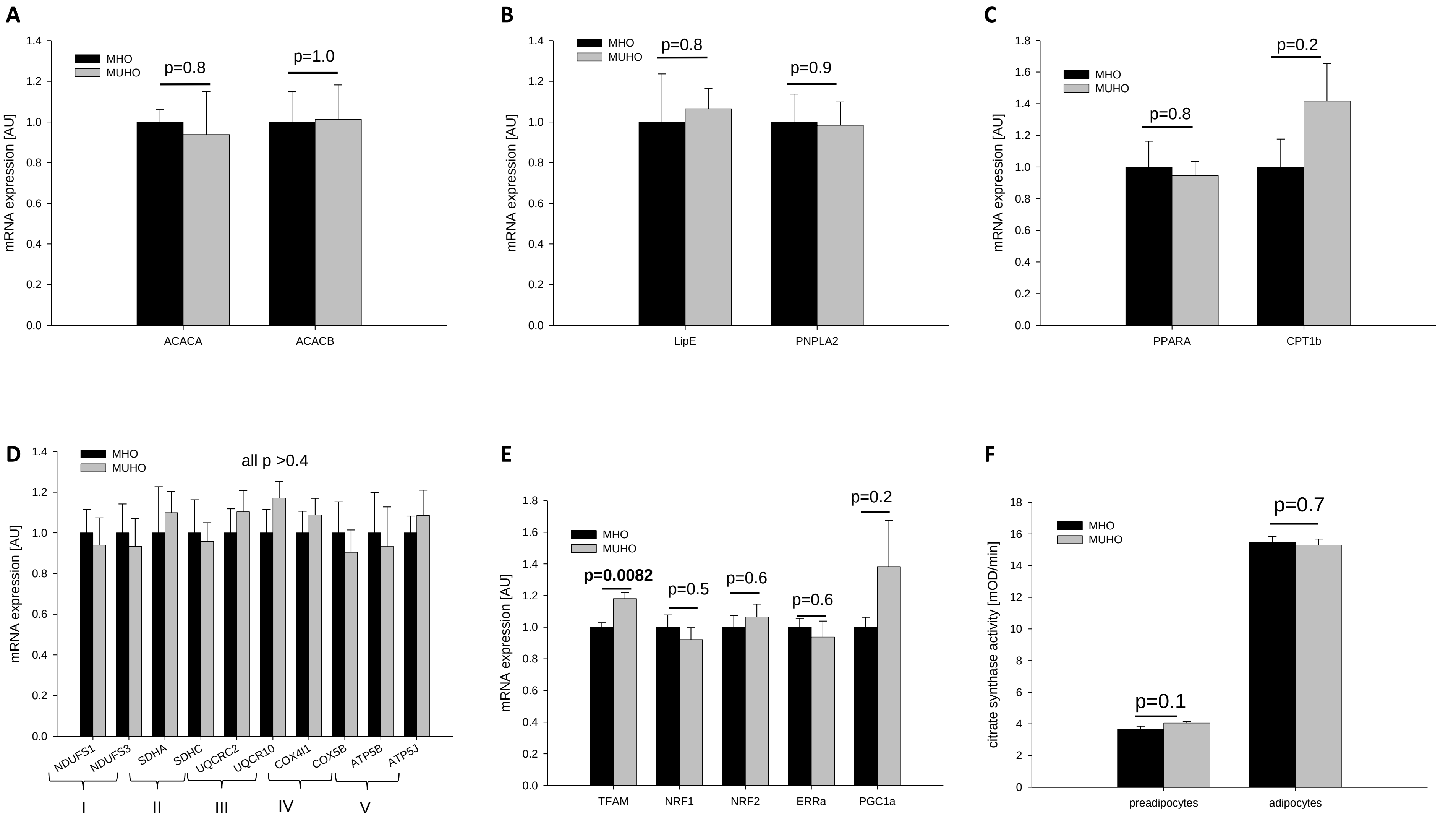
A
MHO
MUHO
p=0.8
p=1.0
mRNA expression [AU]
ACACA
ACACB
B
MHO
MUHO
p=0.8
p=0.9
mRNA expression [AU]
LipE
PNPLA2
C
MHO
MUHO
p=0.8
p=0.2
mRNA expression [AU]
PPARA
CPT1b
D
MHO
MUHO
all p >0.4
mRNA expression [AU]
NDUFS1
NDUFS3
SDHA
SDHC
UQCRC2
UQCR10
COX4I1
COX5B
ATP5B
ATP5J
I
II
III
IV
V
E
MHO
MUHO
p=0.0082
p=0.5
p=0.6
p=0.6
p=0.2
mRNA expression [AU]
TFAM
NRF1
NRF2
ERRa
PGC1a
F
MHO
MUHO
p=0.1
p=0.7
citrate synthase activity [mOD/min]
preadipocytes
adipocytes

Western Blots

fig. S2

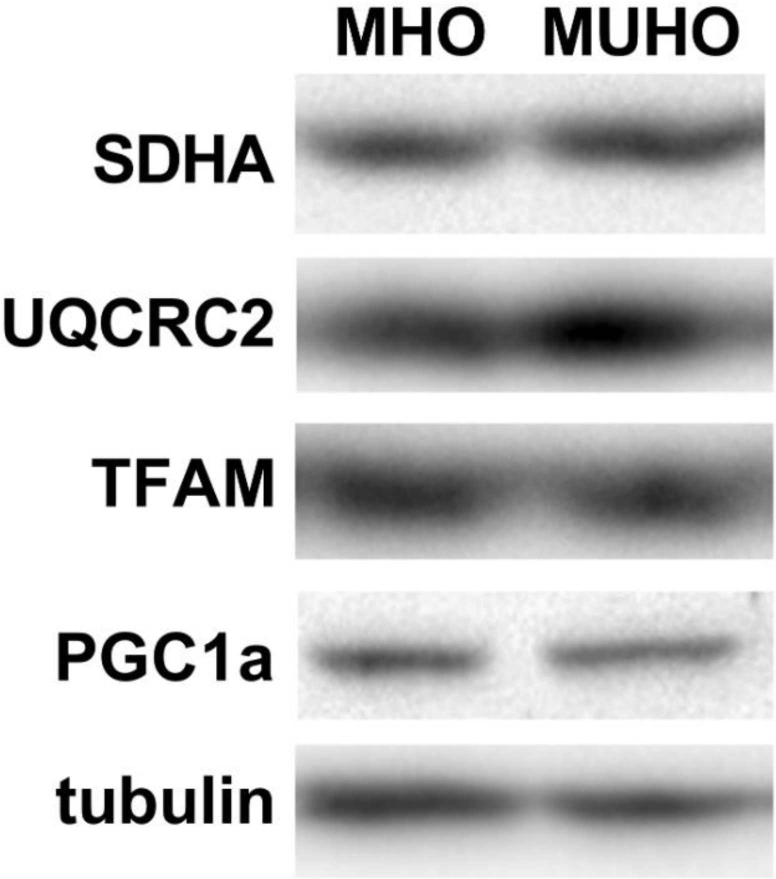

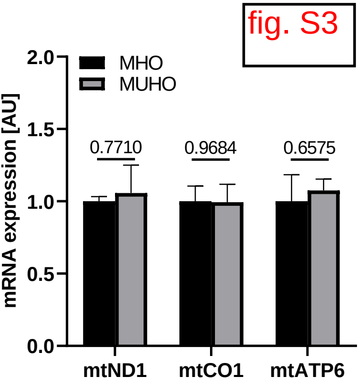
fig. S3
MHO
MUHO
mRNA expression [AU]
2.0
1.5
1.0
0.5
0.0
0.7710
0.9684
0.6575
mtND1
mtCO1
mtATP6

fig. S4

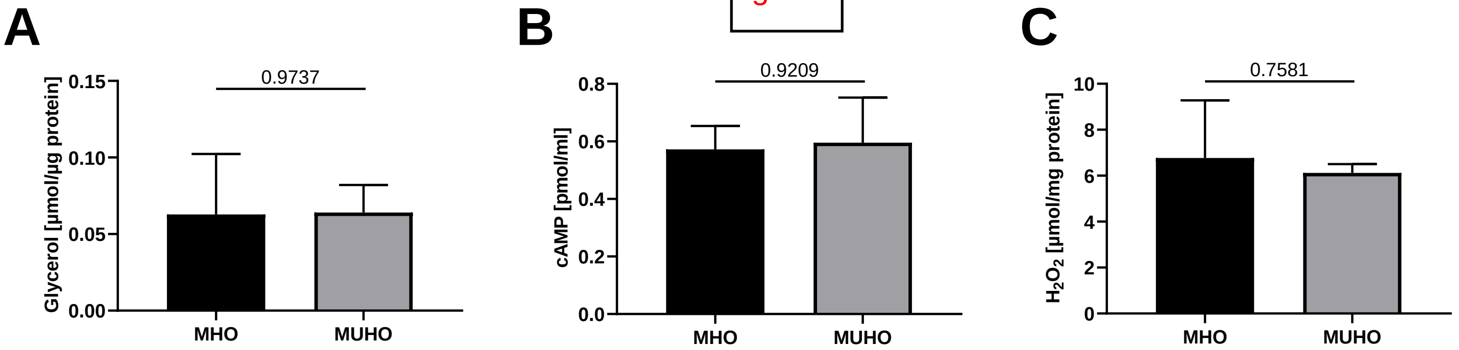
A
Glycerol [µmol/µg protein]
0.15
0.10
0.05
0.00
0.9737
MHO
MUHO
B
cAMP [pmol/ml]
0.8
0.6
0.4
0.2
0.0
0.9209
MHO
MUHO
C
$H_2O_2$ [µmol/mg protein]
10
8
6
4
2
0
0.7581
MHO
MUHO

fig. S5

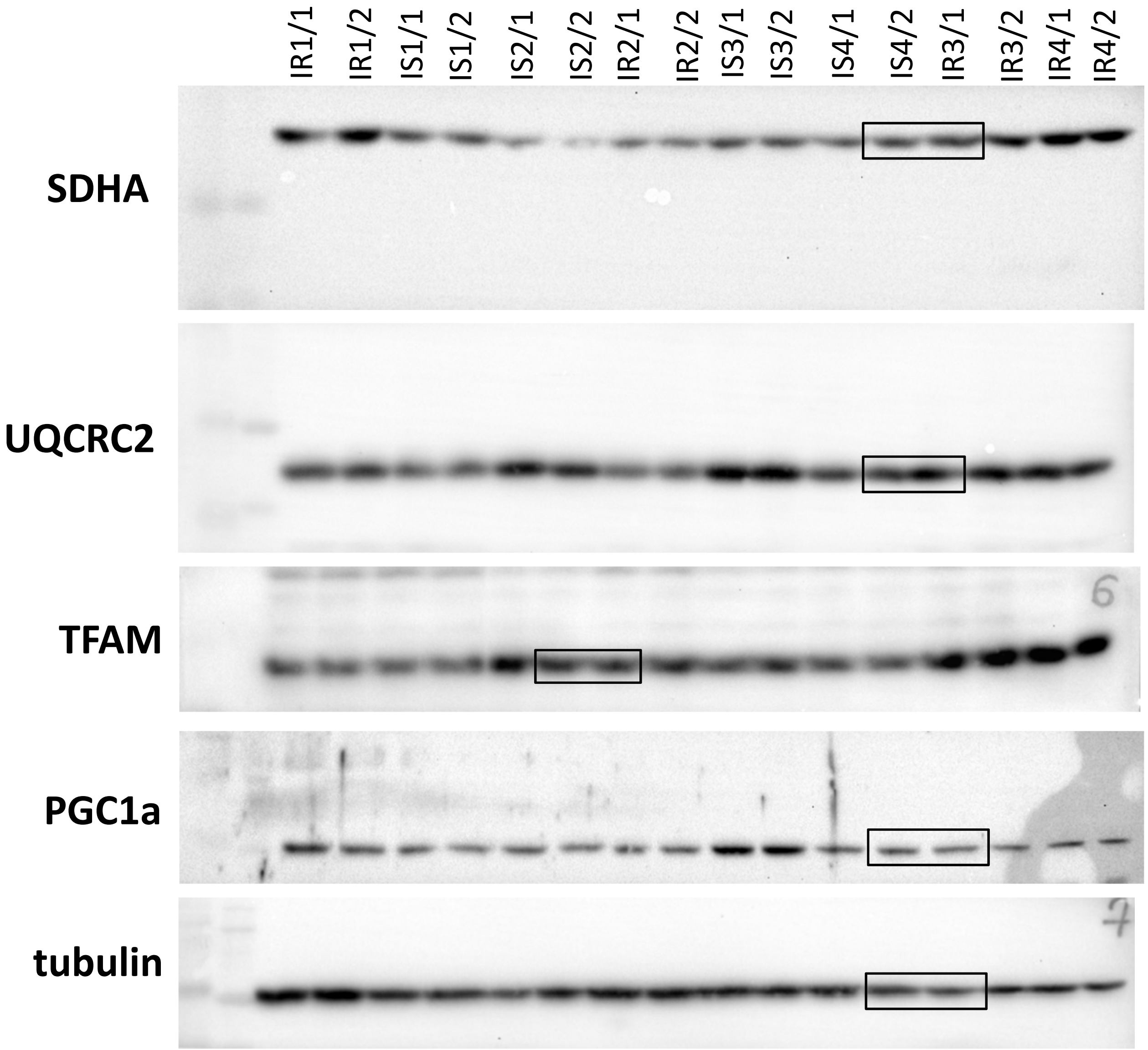
IR1/1
IR1/2
IS1/1
IS1/2
IS2/1
IS2/2
IR2/1
IR2/2
IS3/1
IS3/2
IS4/1
IS4/2
IR3/1
IR3/2
IR4/1
IR4/2
SDHA
UQCRC2
TFAM
6
PGC1a
tubulin
7

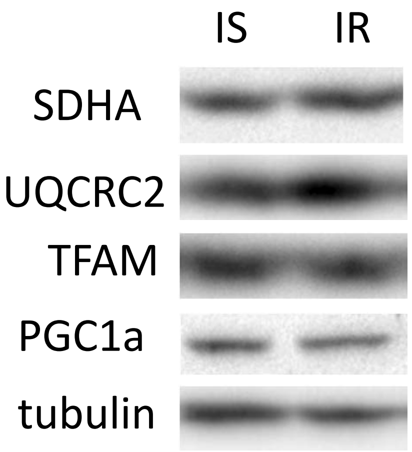
IS
IR
SDHA
UQCRC2
TFAM
PGC1a
tubulin

fig. S6

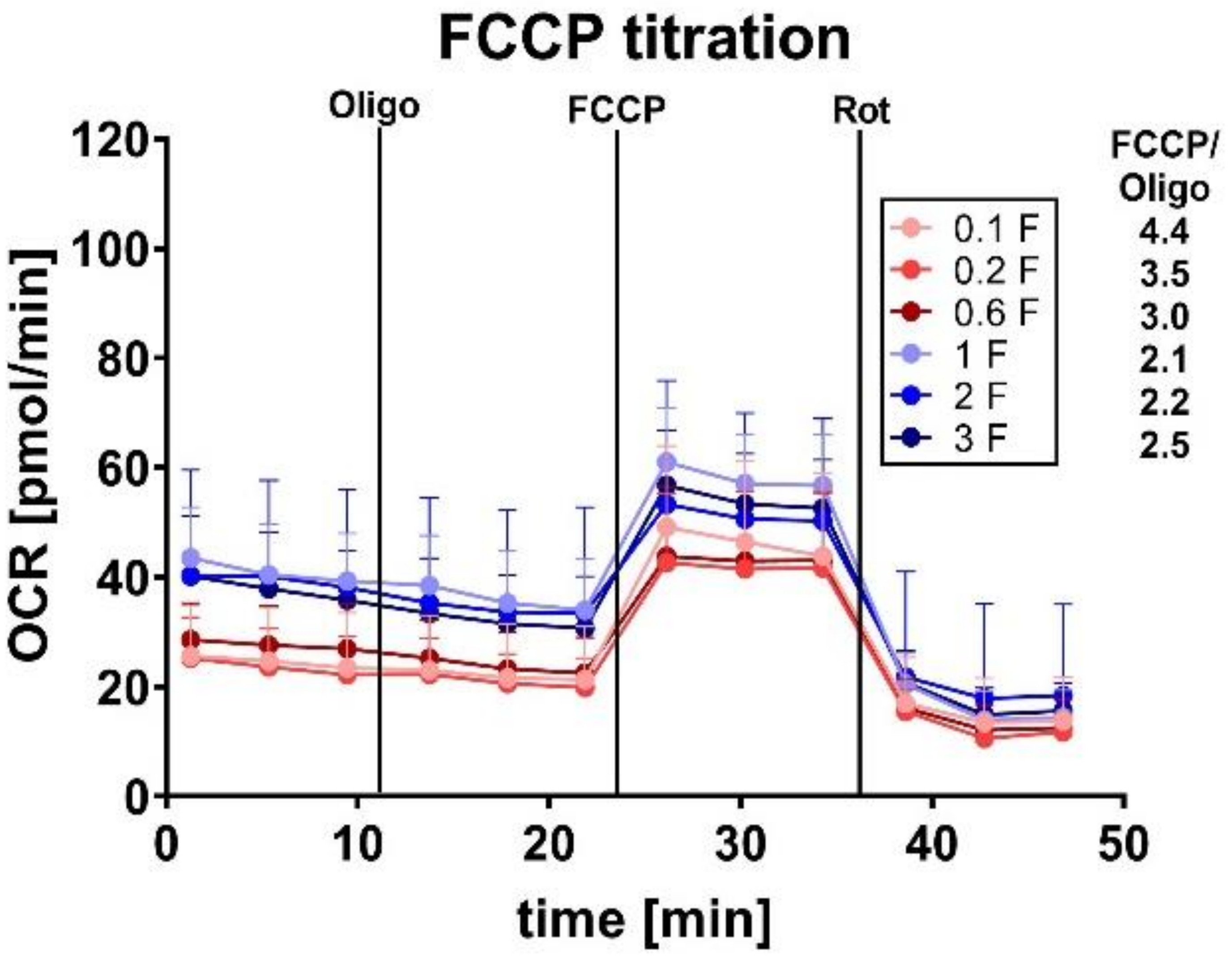
FCCP titration
Oligo
FCCP
Rot
120
100
80
60
40
20
0
OCR [pmol/min]
0
10
20
30
40
50
time [min]
0.1 F
0.2 F
0.6 F
1 F
2 F
3 F
FCCP/
Oligo
4.4
3.5
3.0
2.1
2.2
2.5

| gene | sequence 5' - 3' |
|---|---|
| ACACA up | CATTCCGAGCAAGGGATAAG |
| ACACA do | CAGAGCAGGCTCCAGATGA |
| ACACB do | TGCGGAACATCTCATAGGC |
| ACACB up | TCGGGTCATCGAGAAGGT |
| ATP5B do | ACCAGAATTTCCTGCTCAACA |
| ATP5B up | TGATGAAAGAGGTCCCATCAA |
| ATP5J do | AAGCTCCCTCTCCAGCTCTT |
| ATP5J up | CAGAAACTCTTTGTGGACAAGATTAG |
| COX4I1 do | CAGACAGGTGCTTGACATGG |
| COX4I1 up | TCCACCTCTGTGTGTGTACGA |
| COX5B do | TGTATGGGTCCAGTCCCTTCT |
| COX5B up | TCCATGGCATCTGGAGGT |
| CPT1b do | GAAGGAGGAACCCACTGTTG |
| CPT1b up | CCTGGAAGAAACGCCTGAT |
| ESRRA do (ERRa) | TCAACCACCAGCAGATGAGA |
| ESRRA up (ERRa) | GTGGGCGGCAGAAGTACA |
| LipE do | TTCGTTCCCCTGTTGAGC |
| LipE up | CGCTGGAGGAGTGCTTCTT |
| NDUFS1 do | AAGAAGATAATTGGAACGCAAATC |
| NDUFS1 up | TCTGACACCTTATGCACTGAAGA |
| NDUFS3 do | AACTCATTGAAGCAGGACACCT |
| NDUFS3 up | AGCAGCTCTCAGCTTTTGGA |
| NFE2L2 up (NRF2) | GAGACAGGTGAATTTCTCCCAAT |
| NFE2L2 do (NRF2) | TTTGGGAATGTGGGCAAC |
| NRF1 do | TGGAGGGTGAGATACAGAGGA |
| NRF1 up | TATCCGGAAGAGGCAACAAA |
| PGC1a do | ATAAATCACACGGCGCTCTT |
| PGC1a up | TGAGAGGGCCAAGCAAAG |
| PNPLA2 do | CCCTGCTTGCACATCTCTC |
| PNPLA2 up | CTCCACCAACATCCACGAG |
| PPARa do | GTCCCCGCAGATTCTACATT |
| PPARa up | GAAGCTGTCCTGGCTCAGAT |
| SDHA do | CCAGCGTTTGGTTTAATTGG |
| SDHA up | GGACCTGGTTGTCTTTGGTC |
| SDHC do | CTGCACTCAAAGCAATACCAGT |
| SDHC up | TCCTCTGTCTCCCCACATTAC |
| TFAM do | GAATCAGGAAGTTCCCTCCA |
| TFAM up | GAACAACTACCCATATTTAAAGCTCA |
| UQCR10 do | CTCCAGCTCTGGGCAAAC |
| UQCR10 up | GGGGTGACAGTGGAGTAGAGAA |
| UQCRC2 do | TGCACTTGTGAAATGGTTCTG |
| UQCRC2 up | TGTCCTGACTATAGGATTGGAAAA |
| mtND1 up | AACCTCTCCACCCTTATCACAA |
| mtND1 do | TCATATTATGGCCAAGGGTCA |
| mtCO1 up | CCCTCCCTTAGCAGGGAAC |

| | |
|---|---|
| mtCO1 do | TGAAATTGATGGCCCCTAAG |
| mtATP6 up | GCCGCAGTACTGATCATTCTATT |
| mtATP6 do | GGGTGGTGATTAGTCGGTTG |

Table S1: Primer sequences used for quantitative real-time PCR.
